# STAT3-mediated activation of *miR-21* is involved in down-regulation of TIMP3 and neovascularization in the ischemic retina

**DOI:** 10.18632/oncotarget.21592

**Published:** 2017-10-06

**Authors:** Diana R. Gutsaeva, Menaka Thounaojam, Shubhra Rajpurohit, Folami L. Powell, Pamela M. Martin, Stephanie Goei, Michael Duncan, Manuela Bartoli

**Affiliations:** ^1^ Department of Ophthalmology, Augusta University, Augusta, GA 30912, USA; ^2^ Department of Biochemistry and Molecular Biology, Augusta University, Augusta, GA 30912, USA; ^3^ Department of Internal Medicine, Section of Gastroenterology, Augusta University, Augusta, GA 30912, USA

**Keywords:** ischemic retinopathies, retinal neovascularization, STAT3, miR-21, TIMP3

## Abstract

Retinal neovascularization (RNV) is a sight threatening complication of ischemic retinopathies with limited therapeutic options. The transcription factor signal transducer and activator of transcription 3 (STAT3) has been shown to play a crucial role in promoting RNV. However, manipulating of STAT3 activity can cause significant adverse side effects due to its neurotrophic properties. In this study, we identified microRNA-21 *(miR-21)* as a downstream effector of STAT3 activity in the ischemic retinas and determined its role in promoting RNV through inhibition of its molecular target, the tissue inhibitor of matrix metalloproteinases 3 (TIMP3). Using human retinal endothelial cells (HREC) exposed to hypoxia and a mouse model of oxygen-induced retinopathy (OIR), we found that TIMP3 expression was significantly decreased at both mRNA and protein levels and this paralleled the activation of STAT3 and up-regulation of *miR-21*. Moreover, TIMP3 expression was restored by knockdown of STAT3 or blocking of *miR-21* in HREC, thus, confirming TIMP3 as a downstream target of STAT3/*miR-21* pathway. Finally, in a mouse model of OIR, blockade of *miR-21* by a specific antisense (*a.miR-21*), halted RNV and this effect was associated with rescuing of TIMP3 expression. Our data show that *miR-21* mediates STAT3 pro-angiogenic effects in the ischemic retina, thus suggesting its blockade as a potential therapy to prevent/halt RNV.

## INTRODUCTION

Retinal neovascularization (RNV) is characterized by the inappropriate growth of retinal capillaries which may progress to retinal scarring, detachment and vision loss [1]. This potentially blinding condition is a severe complication of ischemic retinopathies, such as proliferative diabetic retinopathy, retinopathy of prematurity, and retinal vessel occlusion [2, 3].

To date, treatments for RNV involve pan-retinal laser photocoagulation and intravitreal injections of anti-vascular endothelial growth factor (VEGF), however these procedures are not effective in all patients and have potential side-effects [4, 5]. A better understanding of the molecular mechanisms involved in the induction and progression of RNV may provide new clues and allow the identification of more effective therapeutic and diagnostic tools.

Although, RNV occurs in ocular pathologies with different etiologies, some recurrent, common pathogenic features have been identified. These include enhanced production of VEGF [6–9], activation of matrix metalloproteinases (MMPs) [10–12], and up-regulation of pro-inflammatory mediators [9, 13–16]. Several studies have established that the transcription factor signal transducer and activator of transcription 3 (STAT3) is activated in the ischemic retina where it functions as a key modulator of both pro-angiogenic and pro-inflammatory processes [17–20]. Our laboratory and others have demonstrated that STAT3 is a critical mediator of VEGF expression and activity in microvascular endothelial cells [18, 20]. In addition, we have shown that blockade of STAT3 limits RNV [8, 21], thus suggesting that activation of STAT3 represents an important pathogenic hub for RNV. However, the pleiotropic activity of this transcription factor [22], renders its blockade potentially harmful to the neuroretina and moves the attention towards downstream effectors more exclusively mediating its pro-angiogenic and pro-inflammatory activity.

Recent studies have shown that STAT3 is a transcriptional regulator of microRNAs (*miRNA*s) [23–25]. These short noncoding RNAs have emerged as important regulators of post-transcriptional gene silencing by interfering with RNA translation [26, 27]. Increasing body of evidence demonstrates that *miRNA*s are key modulators of different cellular processes and have been implicated in human pathologies including ischemic retinopathies [27–34].

*MiR-21* is one the *miRNAs* transcriptionally regulated by STAT3 [23, 35, 36]. Dysregulated *miR-21* expression has been implicated in a number of human pathologies where inflammation and cell proliferation play a pathogenic role (reviewed in [37]). Moreover, *miR-21* is expressed in retinal microvascular cells [37, 38] and found in human vitreous [39]. Altered *miR-21* expression and function have been recently shown to contribute to the pathogenesis of diabetic retinopathy [40].

*MiR-21* has been involved in angiogenesis and tissue neovascularization [40–43]. Of interest, one *miR-21* gene target is the tissue inhibitor of matrix metalloproteinase 3 (TIMP3) whose angiostatic functions are well-characterized also in retina [44–46].

Based on the above information, we have used loss- and gain-of-function approaches to perform experiments *in vitro* in human retinal endothelial cells (HREC) exposed to hypoxic conditions and *in vivo*, in a murine model of oxygen-induced retinopathy (OIR) to investigate *miR-21* regulation and function in the ischemic retina and its correlation to the STAT3 and TIMP3 pathways.

## RESULTS

### STAT3-dependent *miR-21* up-regulation in HREC in response to hypoxia

**Figure 1:**
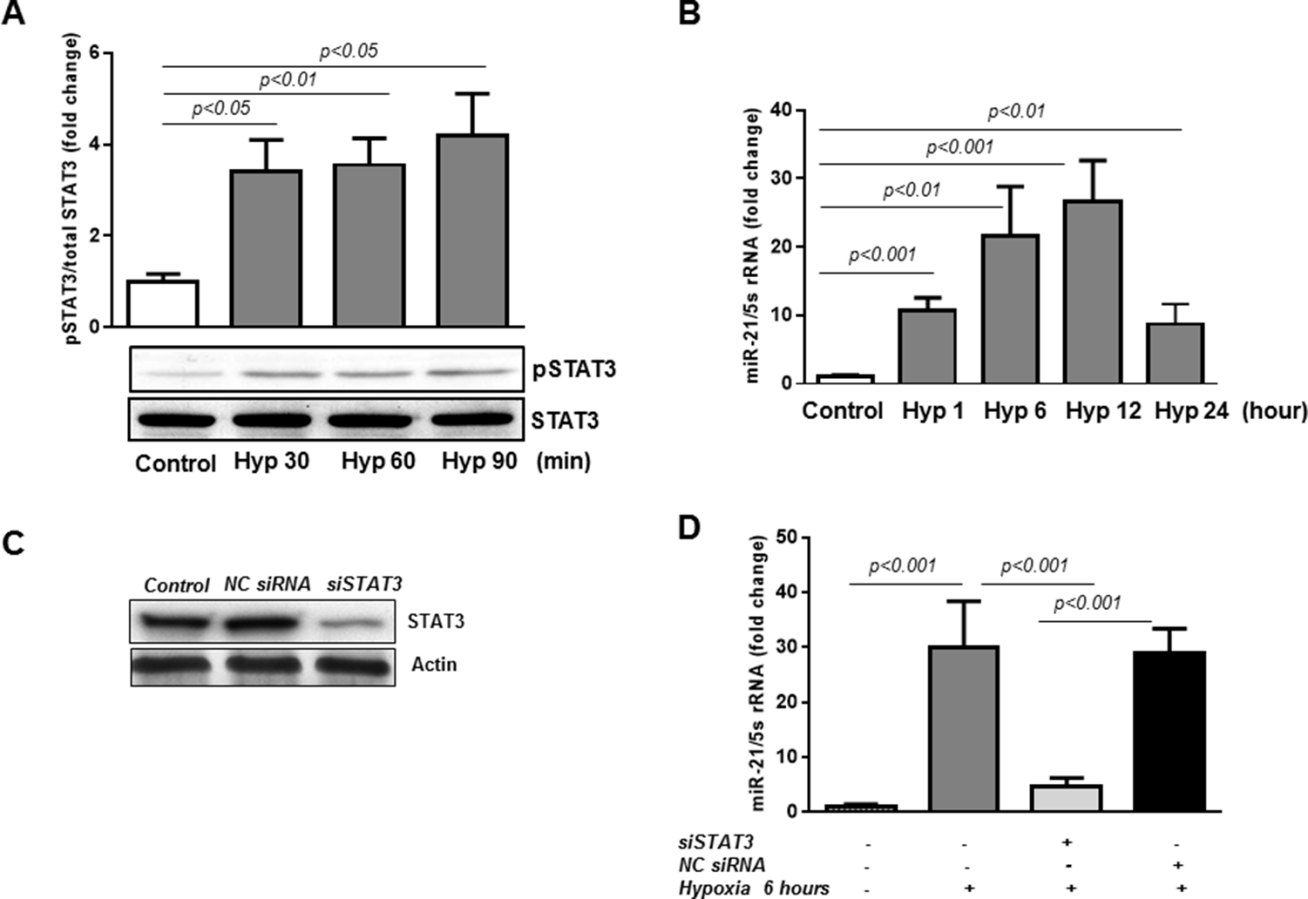
*miR-21* is a down-stream target of STAT3 in HREC in hypoxia **(A)** STAT3 is activated by hypoxia in HREC. Western blotting analysis showing phosphorylation of STAT3 at tyrosine 705 in HREC exposed to hypoxia (pO_2_=2%) for 30-90 min. Blots were subjected to densitometry analysis and the obtained data were analyzed for statistical significance. **(B)** *MiR-21* is up-regulated in HREC in hypoxia. QPCR analysis demonstrating *miR-21* expression in HREC exposed to hypoxia (pO_2_=2%) for 1-12 hours. **(C)** STAT3 protein expression by Western blot showing that STAT3 siRNA (50 nM final concentration), but not *NC* siRNA, decreased total STAT3 protein in HREC by ∼70% assessed 48 hours after transfection. **(D)** Blocking of STAT3 down-regulates *miR-21* expression in hypoxia. QPCR showing *miR-21* expression in HREC transfected with siRNA constructs for STAT3 and *NC* siRNA and exposed to hypoxia (pO_2_=2%) for 6 hours. Values are mean ± SD of four separate experiments.

In the current study, we first confirmed that hypoxia promotes rapid activation of STAT3 in human retinal endothelial cells. As shown in Figure 1A, phosphorylation of STAT3 at tyrosine 705, required for STAT3 activation, was increased more than 3-fold as early as 30 minutes after initiation of hypoxia (pO_2_=2%; p<0.05) and remained elevated for at least 90 minutes (p<0.05). Next, we evaluated the expression levels of *miR-21* in HREC exposed to hypoxia by qPCR. These results revealed that *miR-21* expression closely followed the pattern of STAT3 phosphorylation/activation with significant increase observed one hour after the onset of hypoxia (Figure 1B; p<0.001) and progressive increase with maximal expression at 12 hours (p<0.001). At 24 hours of hypoxia *miR-21* levels decreased compared to 12 hours (Figure 1B).

To determine the specific role of STAT3 in transcriptional regulation of *miR-21* in hypoxic retinal endothelial cells, HREC were transfected with STAT3-specific siRNAs (Figure 1C-1D). Western blotting analysis demonstrated that in HREC transfected with STAT3-specific siRNAs STAT3 protein levels were reduced by ∼70%, whereas the non-targeted (*NC*) siRNAs had no effect (Figure 1C). In addition, transfection of HREC with STAT3-specific siRNAs significantly downregulated the expression of *miR-21* induced by hypoxia (pO_2_=2%; 6 hours), whereas transfection of the cells with *NC* siRNAs had no effects (Figure 1D). These data show that *miR-21* expression in HREC exposed to hypoxia is STAT3-dependent.

### *MiR-21* promotes HREC *in vitro* tube formation

**Figure 2:**
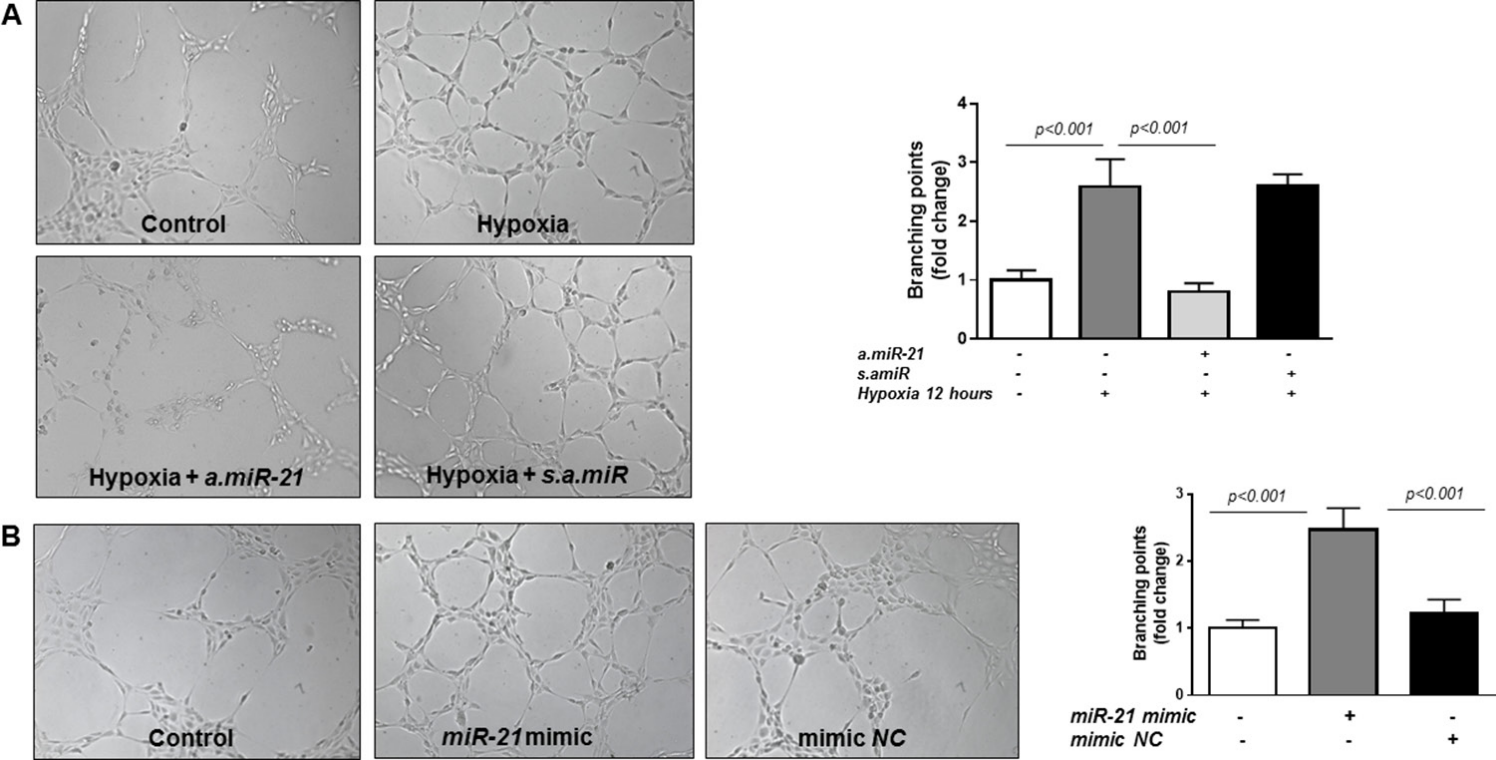
Matrigel tube formation assay in HREC **(A)** Representative images of tube formation and quantification of branching points in HREC transfected with *a.miR-21* (50 nM final concentration) and *s.amiR* and exposed to hypoxia (pO_2_=2%) for 12 hours. **(B)** Representative images of tube formation and quantification of branching points in HREC transfected with *miR-21* mimic (50 nM) or mimic *NC* for 48 hours (B). Tube formation was observed under an inverted microscope and the images were captured with a digital camera. Representative images and analysis of three independent experiments. Original magnification, x10.

Despite the overwhelming evidence suggesting its pro-angiogenic activity, *miR-21* has shown to have also anti-angiogenic effects [47]. Therefore, here we explored the effects of altering *miR-21* expression in HREC in a matrigel tube formation assay. The extent of network formation was quantified by determining the number of branching points. Figure 2A shows that transfection of HREC with an antisense inhibitor of *miR-21* (*a.miR-21*) significantly decreased hypoxia-induced formation of network-like structures in the matrigel assay compared to cells transfected with the scrambled inhibitor negative control (*s.amiR*). Furthermore, overexpression of *miR-21* mimic in HREC (Figure 2B) promoted tube formation as demonstrated by increase in the branching point number (2.5-fold) as compared to non-transfected control HREC or cells transfected with mimic negative control (*NC*) (Figure 2B). Thus, these data indicate that *miR-21* exerts pro-angiogenic activity in HREC.

### STAT3/*miR-21* axis is involved in TIMP3 regulation in hypoxia

**Figure 3:**
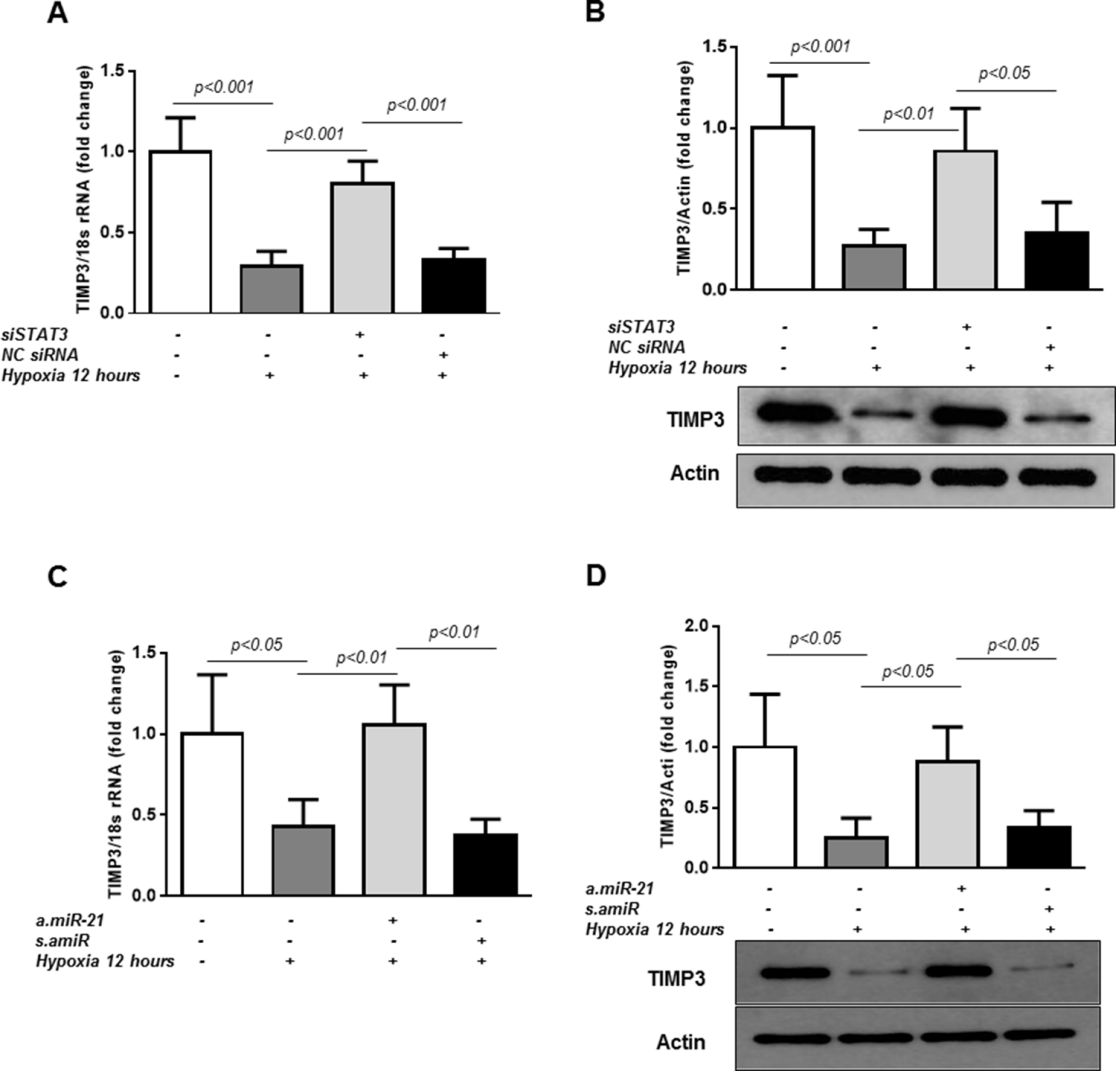
STAT3/*miR-21* axis is involved in TIMP3 expression in hypoxic HREC **(A)** QPCR of TIMP3 mRNA expression in control untreated HREC and cells transfected with STAT3-specific siRNA (50 nM final concentration) or negative control siRNA (*NC*) and exposed to hypoxia (pO_2_=2%) for 12 hours. **(B)** Western blot showing expression of TIMP3 in HREC transfected with STAT3-specific siRNA or *NC* siRNA. Blots were subjected to densitometry analysis and the obtained data were analyzed for statistical significance. **(C)** QPCR of TIMP3 mRNA expression in HREC transfected with *a.miR-21* (50 nM final concentration) and *s.amiR* and exposed to 2%O_2_ hypoxia for 12 hours. **(D)** Western blot showing expression of TIMP3 in HREC transfected with *a.miR-21* and *s.amiR*. Blots were subjected to densitometry analysis and the obtained data were analyzed for statistical significance. Values are mean ± SD of four separate experiments.

To determine whether STAT3/*miR-21* pathway is implicated in the regulation of TIMP3 in retinal endothelial cells in hypoxic conditions, TIMP3 expression was first assessed in HREC exposed to hypoxic conditions with or without transfection of STAT3-specific siRNAs. As shown in Figure 3, TIMP3 mRNA (Figure 3A) and protein (Figure 3B) levels were suppressed in HREC challenged with 12 hours of hypoxia (pO_2_=2%; p<0.001 vs control untreated cells). Knockdown of STAT3 with specific siRNAs as demonstrated in Figure 3A-3B rescued TIMP3 mRNA and TIMP3 protein levels in hypoxic HREC as compared to non-transfected hypoxic cells. On the contrary, in HREC transfected with *NC* siRNAs and exposed to hypoxia the levels of TIMP3 mRNA and protein remained unchanged compared to non-transfected hypoxic cells (Figure 3A-3B).

TIMP3 has previously been validated as *miR-21* target in different cells and experimental settings [23, 42, 48]. To confirm the role of *miR-21* in STAT3-dependent TIMP3 suppression in hypoxia, TIMP3 mRNA and protein levels were measured in HREC transfected with *miR-21* inhibitor, *a.miR-21*, or a scrambled control, *s.amiR*, after 12 hours of hypoxia. Transfection of the cells with *a.miR-21* rescued hypoxia-mediated decrease in TIMP3 mRNA (Figure 3C) and protein (Figure 3D) as compared to untreated cells. No changes in TIMP3 mRNA or protein levels were observed in hypoxic HREC transfected with *s.amiR* as compared to non-transfected hypoxic cells (Figure 3C-3D).

**Figure 4:**
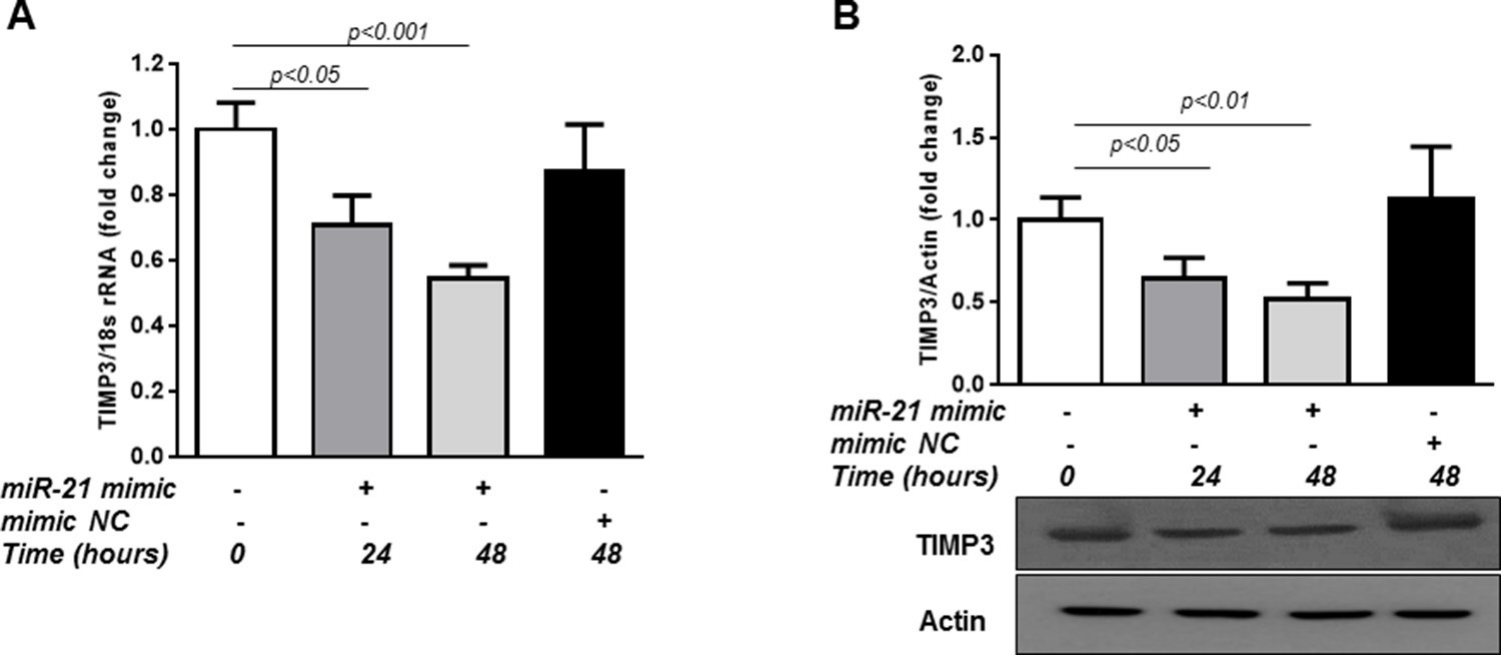
Overexpression of *miR-21* up-regulates TIMP3 expression in HREC **(A)** QPCR of TIMP3 mRNA expression in control HREC and HREC transfected with *miR-21* mimic (50 nM) for 24 and 48 hours or mimic *NC*. **(B)** Western blot showing expression of TIMP3 in HREC transfected with *miR-21* mimic or mimic *NC*. Values are mean ± SD of four separate experiments.

Finally, we determined whether overexpression of *miR-21* would be sufficient to reduce TIMP3 levels in retinal endothelial cells. *MiR-21* overexpression was achieved by transfection of HREC with *miR-21* mimic for 24 and 48 hours. As demonstrated in Figure 4, TIMP3 mRNA (Figure 4A; p<0.05 and p<0.001 for 24 and 48 hours respectively) and protein levels (Figure 4B; p<0.05 and p<0.01 for 24 and 48 hours respectively), were reduced in *miR-21* mimic transfected cells compared to control untreated cells whereas transfection of the cells with mimic *NC* had no effect. Overall, these data strongly indicate that STAT3/*miR-21* axis is involved in TIMP3 downregulation in the hypoxic milieu.

### STAT3/*miR-21* axis is up-regulated and TIMP3 is suppressed in retinas of OIR mice

**Figure 5:**
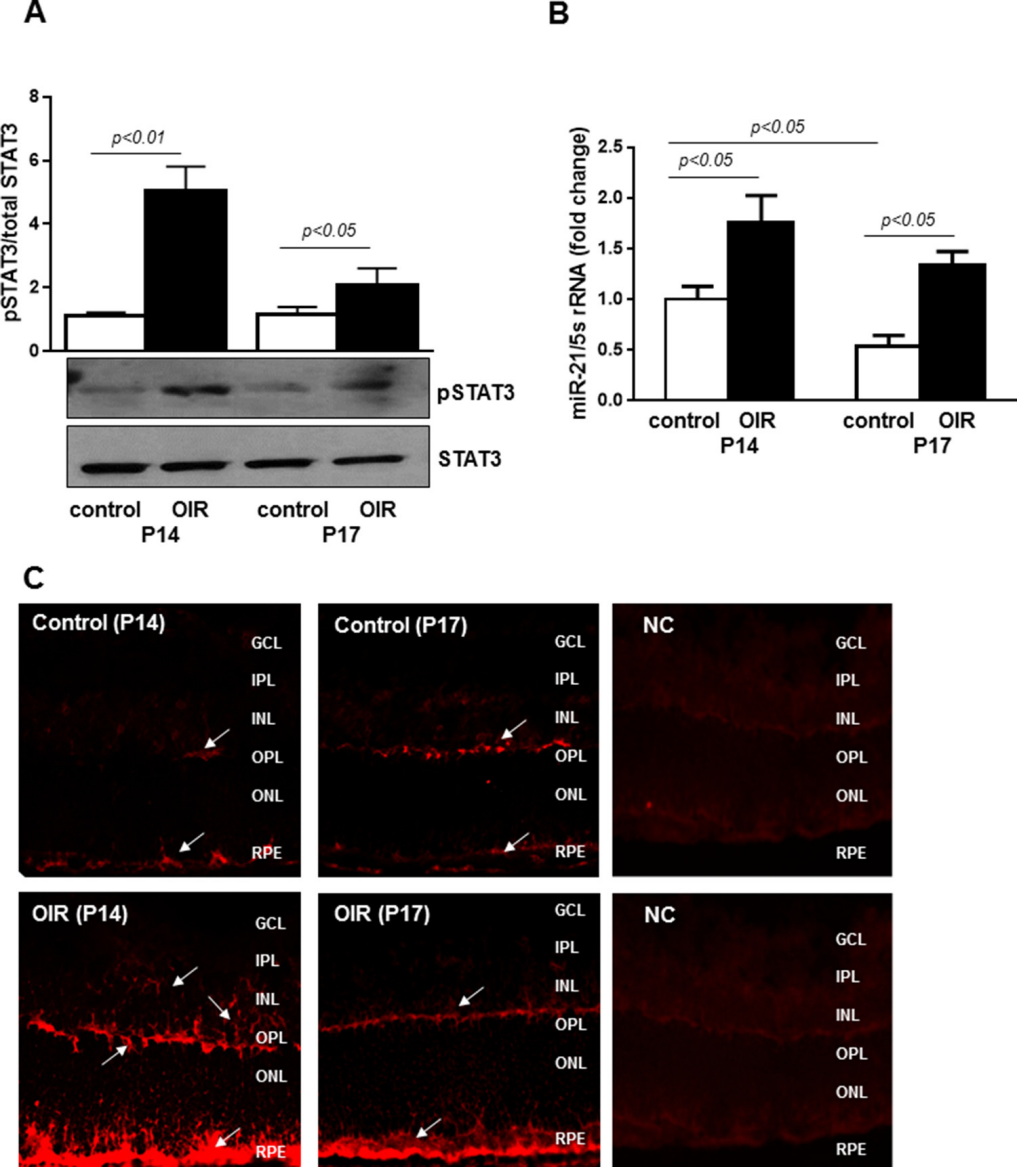
STAT3 and *miR-21* are activated in retinas of OIR mice **(A)** Western blotting analysis showing phosphorylation of STAT3 at tyrosine 705 in retinas of control and OIR mice at P14 and P17. Blots were subjected to densitometry analysis and the obtained data were analyzed for statistical significance. **(B)** QPCR analysis demonstrating *miR-21* expression in retinas of control and OIR mice at P14 and P17. Values are mean ± SD (n=5 retinas per group). **(C)** Representative images of *miR-21* ISH in retinas from OIR (lower images) and control (upper images) mice at P14 and P17 (n=4 retinas per group). Arrows indicate expression of *miR-21* in the INL, OPL, and RPE cell layers. Original magnification, x20.

To validate the effects of *miR-21 in vivo*, we employed a well-characterized model of pathological angiogenesis, the mouse model of OIR. We assessed STAT3 activation and *miR-21* expression in OIR mice focusing on postnatal days 14 and 17 (P14 and P17), time points corresponding to progressive vascular regrowth and neovessel formation. Western blot analysis confirmed that phosphorylation of STAT3 was increased in retinas of OIR mice at P14 and P17 when compared with normal age-matched control mice (Figure 5A). To determine the status of *miR-21* in the ischemic retina, we measured, by qPCR, *miR-21* expression in retinas of control age-matched mice and mice subjected to OIR. As shown in Figure 5B, retinal levels of *miR-21* in OIR mice at P14 and P17 was significantly elevated compared to age-matched control mice (∼1.7 and 2.4 fold at P14 and P17, respectively). Consistent with the qPCR analysis, *in situ* hybridization (ISH), using an anti-*miR-21* probe, confirmed the up-regulation of *miR-21* transcripts in retinas of mice subjected to OIR at both P14 and P17 (Figure 5C; lower panels) compared to age-matched control mice (Figure 5C; upper panels). ISH further revealed that *miR-21* expression is localized in the inner nuclear (INL), outer plexiform (OPL), and retinal pigment epithelium (RPE) cell layers (Figure 5C; arrows) with the maximum signal observed in OIR retinas at P14.

**Figure 6:**
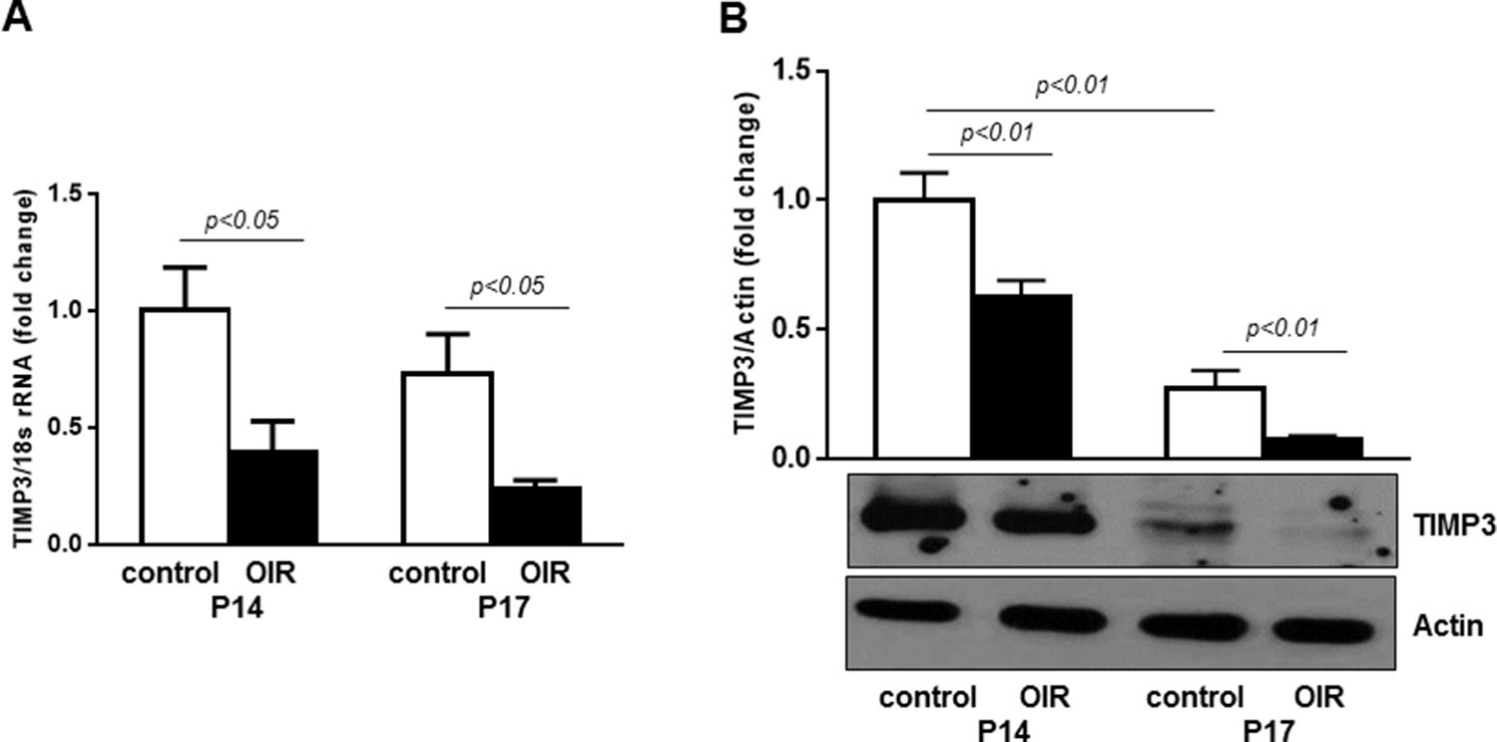
TIMP3 is downregulated in retinas of OIR mice **(A)** QPCR of TIMP3 mRNA expression in retinas of control and OIR mice at P14 and P17. **(B)** Western blot showing expression of TIMP3 in retinas of OIR and age-matched control mice. Blots were subjected to densitometry analysis and the obtained data were analyzed for statistical significance. Values are mean ± SD (n=5 retinas per group).

Next, we assessed the expression of TIMP3 in retinas of control mice and mice following OIR by qPCR and immunoblotting analyses. Figure 6A shows that retinal TIMP3 mRNA expression was significantly down-regulated in OIR mice at postnatal days P14 and P17 compared to control mice of the same age (∼2.5 and 3.1 fold at P14 and P17, respectively). The decrease in TIMP3 mRNA expression was followed by reduction in TIMP3 protein as shown in Figure 6B (p<0.01 vs respective control mice).

### Intraorbital delivery of *miR-21* inhibitor rescues TIMP3 expression and decreases retinal vascular regrowth and pathological neovascularization

**Figure 7:**
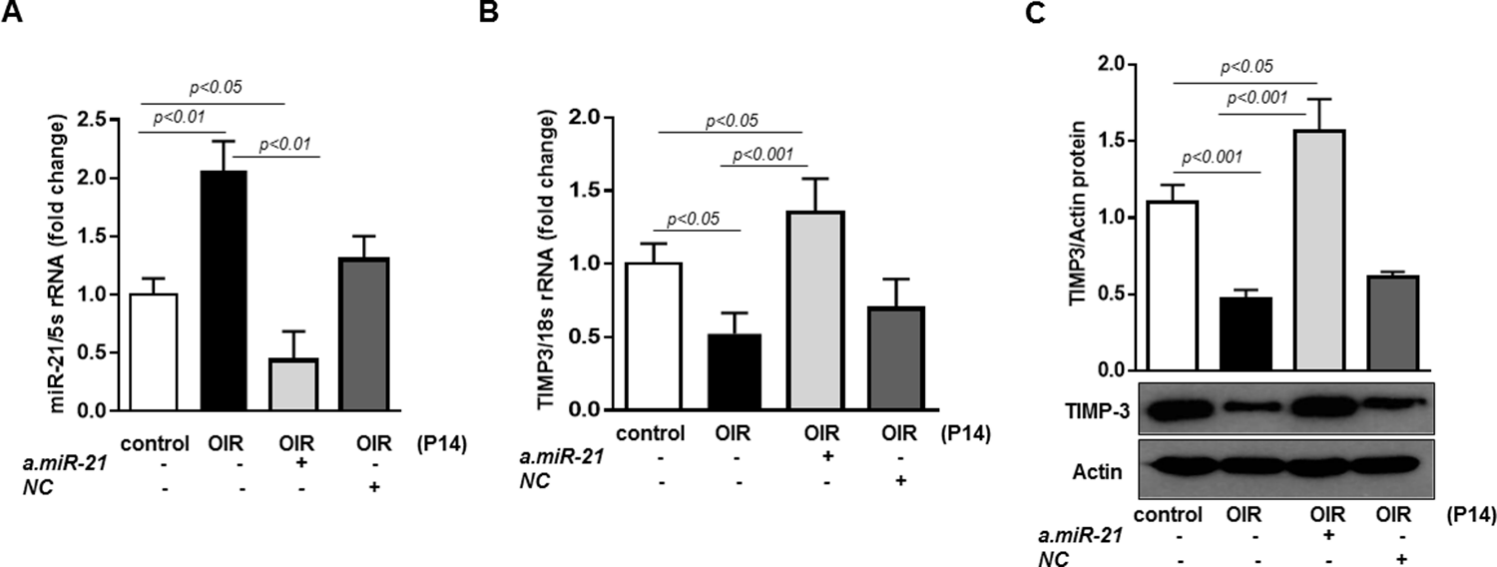
Intraorbital delivery of miR-21 inhibitor recovers TIMP3 expression in retinas of OIR mice **(A)** *MiR-21* expression in retinas of control mice and OIR mice at P14 injected with *miR-21* inhibitor (*a.miR-21*) or *NC* LNA. **(B)** QPCR of TIMP3 mRNA expression in retinas of control mice and OIR mice at P14 injected with a.*miR-21* or *NC* LNA. **(C)** Western blot showing expression of TIMP3 in retinas of control mice and OIR mice at P14 injected with *a.miR-21* or *NC* LNA. Blots were subjected to densitometry analysis and the obtained data were analyzed for statistical significance. Values are mean ± SD (n=5 retinas per group).

We blocked retinal endogenous *miR-21* in mice subjected to OIR by intraorbital injection of seed-targeting miRCURY *miR-21* LNA inhibitor (*a.miR-21*) at postnatal day 11. To confirm that LNA inhibitor down-regulated retinal *miR-21*, we measured *miR-21* expression in retinas of treated and un-treated OIR mice at P14. As demonstrated in Figure 7A, *a.miR-21* decreased levels of *miR-21* in retinas of OIR mice compared to untreated OIR mice. In parallel, upon *miR-21* inhibition, TIMP3 mRNA and TIMP3 protein (Figure 7B-7C, respectively) were induced in OIR mice at P14 compared to untreated OIR mice and OIR mice injected with *NC*. Noticeably, blocking of *miR-21* in OIR mice down-regulated the levels of *miR-21* to levels below than those observed in control mice and up-regulated TIMP3 expression to levels higher than those observed in control P14 mice. This data validates the efficacy of the treatment and suggest that basal *miR-21* expression is involved in TIMP3 regulation also in normal retina.

**Figure 8:**
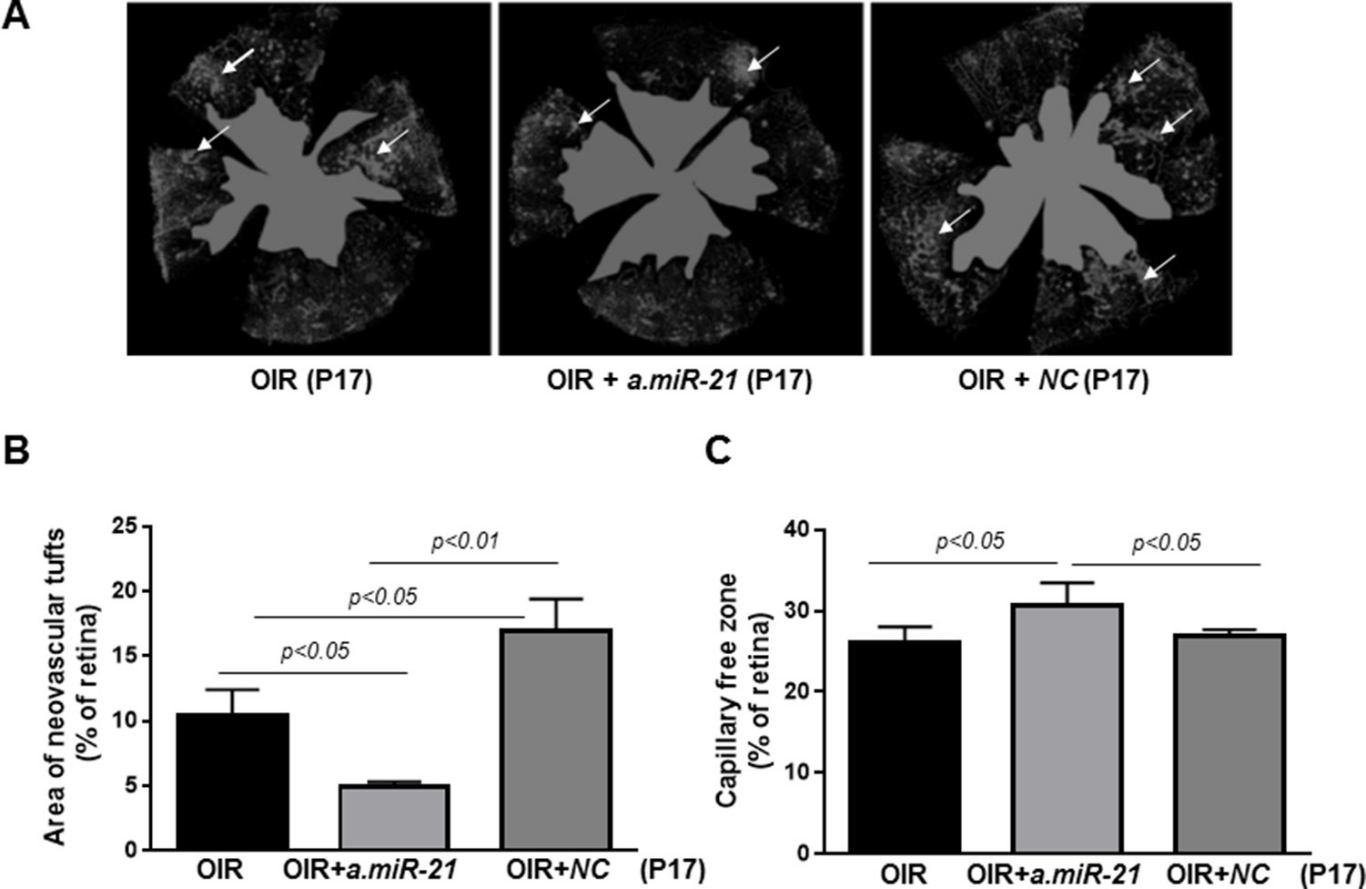
Intraorbital delivery of *miR-21* inhibitor reduces pathological neovascularization in retinas of OIR mice **(A)** Representative flatmounted retinas of OIR mice at P17 injected with *a.miR-21* or *NC* LNA stained with isolectin B4 to identify areas of neovascularization (arrows) and avascular zones (green). **(B-C)** Histograms representing the results of morphometric analysis of retinal flatmounts measuring neovascular tufts (B) and the capillary-free areas (C). Values are mean ± SD (n=4 retinas per group).

Finally, to determine whether blocking of *miR-21* affects RNV, we assessed vascular density and distribution in the retinas of OIR mice at P17 by conducting morphometric analysis of retinal flatmounts stained with Texas Red–isolectin B4 (Figure 8). Quantification of the observed changes revealed that blocking of *miR-21* significantly decreased the number of neovascular tufts in *a.miR-21* treated OIR mice compared to untreated OIR mice (Figure 8B). Some increase in the area of neovascular tufts was observed in OIR mice injected with *NC* LNA as compared to untreated and *a.miR-21* treated OIR mice (Figure 8B). Blocking of *miR-21* resulted in a larger avascular area in *a.miR-21* treated OIR mice compared to untreated or *NC* LNA-treated OIR mice (Figure 8C).

## DISCUSSION

We have identified *miR-21* as a down-stream effector of STAT3 in promoting pathological neovascularization in the ischemic retina. Using loss- and gain-of-function approaches, we provide the evidence that in HREC exposed to hypoxic condition the activation of STAT3 results in *miR-21* gene transcription and consequent suppression of its target gene TIMP3. Moreover, blockade of *miR-21*, reduces the angiogenic potential of endothelial cells *in vitro* and diminishes neovascular tufts formation in the ischemic retina. The obtained data, therefore, indicate that STAT3/*miR-21* axis contributes to RNV.

STAT3 has shown to play a critical role in induction and progression of RNV affecting the expression and activity of VEGF and inflammatory cytokines [17–21]. In agreement with previous findings, STAT3 was found to be rapidly activated in HREC challenged with hypoxia and in retinas of OIR mice during the neovascular stages (P14 and P17).

*MiR-21*, which is located on chromosome 17q23.2 within the protein-coding region of the transmembrane protein 49 [49], is among several *miRNAs* that have been shown to be transcriptionally activated by STAT3 [25, 35]. Profiling studies showed that *miR-21* is one of 28 highly expressed *miRNA*s in endothelial cells, suggesting a contributing role of this *miRNA* to vascular homeostasis [reviewed in [30, 50]]. Consistent with these reports, we detected significant baseline levels of *miR-21* expression in HREC and in retinas of control mice at postnatal days 14 and 17. A potential homeostatic function of *miR-21* is supported also by the presence of this *miRNA* in normal human vitreous [39]. In addition, we have observed a modest, but statistical significant difference in *miR-21* expression in normal mice between postnatal age 14 and 17, suggesting that the retinal expression of *miR-21* is developmentally regulated.

TIMP3 biological function is primarily attributed to its ability to control the activity of MMPs [42, 51–54] and sheddases such as the TNFα-convertase [55] and to inhibit VEGF-VEGFR2 interaction [45]. TIMP3 has been shown to be a negative modulator of choroidal neovascularization [54, 56, 57] and RNV [44, 46]. TIMP3 has shown to be a *miR-21* gene target [23, 48, 58]. In the current study, we showed that inhibition of STAT3 and *miR-21* rescued TIMP3 expression in hypoxic HREC and in *a.miR-21*-treated OIR mice, therefore, confirming the importance of the STAT3/*miR-21* axis in suppression of TIMP3 during RNV. It is important to note that inhibition of *miR-21* in retinas of OIR mice resulted in an elevation of its expression above the levels observed in control mice, thus, underscoring *miR-21* regulatory activity also on retinal TIMP3 basal levels. In parallel to up-regulation of TIMP3, inhibition of *miR-21* in OIR, at P17, resulted in a significant reduction of the neovascular tuft areas and augmentation of the capillary-free zone.

These results are in agreement with other reports demonstrating pro-angiogenic activities of *miR-21 in vivo* [40, 43, 59, 60] and *in vitro* systems [42, 43, 60–62]. Interestingly, *miR-21* acted as a negative modulator of angiogenesis in choroidal neovascularization [47]. The discrepancy between this study and ours could be attributed to different properties of choroidal and retinal endothelial cells.

In summary, our current findings provide the evidence that STAT3/*miR-21* axis represents a central epigenetic switch implicated in RNV.

## MATERIALS AND METHODS

### Animal model of retinal neovascularization and treatment protocols

All the animal procedures were performed in accordance with the statement of the Association for Research in Vision and Ophthalmology (ARVO) for the humane use of animals in vision science and with protocols approved by Augusta University (AU). C57Bl/6J mice were purchased from Jackson laboratories (Bar Harbour, ME) at 2 months of age and kept in AU animal facility with a light cycle of 12 hours and fed ad libitum. RNV was induced in a mouse model of OIR according to the protocol of Smith et al. [63]. In this model, on postnatal day 7 (P7), newborn mice are placed, along with their dam, into a custom-built chamber in which the partial pressure of oxygen is maintained at 75%. After 5 days, the mice are brought to room air and kept until P17. In this model, neo-vascularization forms from P13 through P17 in response to hypoxic conditions in the retina and is quantified at P17, when pathological response is at a maximum. Age-matched C57Bl/6J mice kept at room air are used as controls. To block *miR-21*, OIR mice received at P11, while still in the oxygen chamber, intraorbital injections of 1 μl of seed-targeting miRCURY Locked Nucleic Acid (LNA) inhibitor (*a.miR-21*; *mmu-miR-21a-5p*; Exiqon, Woburn, MA) or LNA inhibitor control (*NC;* Exiqon) in the left eye (3 μg each). The mice were sacrificed by an overdose of anesthetic at P14 and P17. The eyeballs and retinas were excised and processed for imaging or molecular analysis. 2-3 sets of six pups each were used for each treatment group.

### Morphometric analysis

Retinal vessel growth and distribution were assessed in flatmounted retinal preparations labeled with biotinylated isolectin B4 (Griffonia simplicifolia) and Texas Red–conjugated avidin D [21]. Images were captured with a fluorescent microscope (Zeiss Axioplan 2; Carl Zeiss, Thornwood, NY) equipped with AxioVision 4.6.3.0 software. Areas of vaso-obliteration and capillary tufts formation were analyzed using Adobe PhotoShop CS6 according to Connor et al. [64]. The ratio of the non-perfused area and tufts to the entire retinal area was determined. The morphometric analysis was performed in a masked fashion.

### *In situ* hybridization (ISH)

ISH was performed on frozen retinal sections fixed in 4% paraformaldehyde. *miRNAs* were un-masked by incubation with proteinase K for 30 minutes. Slides were incubated overnight at 58°C with a double 5’- and 3’-digoxigenin (DIG)-labeled miRCURY LNA™ probe for *miR-21* (Exiqon). Slides were washed in 2x, 1x and 1x concentrations of sodium citrate buffer at 58°C, 53°C, and 37°C, respectively, followed by one hour incubation with anti-DIG (Roche Diagnostics, Indianapolis, IN) and mounting with Fluoromount (Fisher Scientific, Pittsburg, PA). Images were captured by fluorescent microscopy. Control sections probed with scrambled *mRNA* control (5`- and 3`-DIG-labeled) were used as a negative control (NC).

### Cell culture and hypoxia treatment

HREC were purchased from Cell Systems Corporation (Kirkland, WA) and cultured in a growth medium at 37°C in a humidified atmosphere of 5% CO_2_ in air as suggested by the manufacturer. HREC were used between passages 3-7. Prior to all experiments, HREC monolayers were serum starved for 10-12 hours. Hypoxia was achieved by exposing HREC for 0.5-12 hours to a gas mixture (5% CO_2_-balanced N_2_) to obtain 2% O_2_ in a PRO-OX 110-sealed hypoxia chamber provided with oxygen sensor (BioSperix Limited, Lacona, NY).

### Cell transfection

HREC were plated at a density 4.0-5.0 × 10^5^ into 60 mm plates. Transfection of cells with STAT3 silencer pre-designed small interfering RNA (siRNA; 50 nM; Ambion Waltham, MA) or negative control siRNA (NC; Ambion) constructs, the *miR-21* mimic (miScript *hsa-miR-21-5p mirVana*™ mimic; 50 nM; Ambion) or the *miRNA* mimic negative control (*NC*; Ambion), *hsa-miR-21-5p mirVana*™ inhibitor (*a.miR-21*; 50 nM; Ambion) or scrambled *anti-miR™miRNA* inhibitor negative control (*s.amiR*; Ambion) was performed using Lipofectamine 2000 (Invitrogen) and Lipofectamine RNAiMax Transfection Reagent (Invitrogen, Carlsbad, CA) according to the manufacturer’s instruction. Cells were harvested 24-48 hours after transfection for analyses by qPCR and Western blotting.

### Reverse transcription real-time PCR and *miRNA* assay

Total *RNA* was isolated from retinas and HREC using TRI Reagent (Sigma-Aldrich, St. Louis, MO) according to the manufacturer’s protocol. cDNA was prepared using iScript™cDNA Synthesis Kit (Bio-Rad, Hercules, CA). Amplification of TIMP3 Forward 5`- ACTGCAAGATCAAGTCCTGCT-3`, Reverse 5`-AGGCGTAGTGTTTGGACTGG-3` *mRNA* was performed using Power SYBR green PCR master mix (Applied Biosystems, Foster City, CA). The conditions used for the PCR were as follows: 95°C for 3 min (1 cycle) and 94°C for 20 sec, 55°C for 30 sec, and 72°C for 40 sec (40cycles). The relative *mRNA* abundance was determined by normalizing to 18s ribosomal RNA (rRNA) using the 2Ct method (Ct refers to the threshold value). *MiRNAs* were isolated using a miRCURY LNA™ Universal RT Kit (Exiqon, Woburn, MA) and cDNA was prepared using a Universal cDNA Synthesis Kit (Exiqon) according to the manufacturer’s instructions. QPCR was performed using specific *miR-21* primer, 5' UAGCUUAUCAGACUGAUGUUGA 3 (Qiagen, Germantown, MD), and the ExiLENT SYBR® Green PCR Master Mix (Exiqon). The conditions used for qPCR were as follows: 95°C for 10 min (1 cycle), 95°C for 10 sec, 60°C for 1 min (40 cycles). The thermal cycler StepOne™ Real-Time PCR System (Applied Biosystems; Foster City, CA) was used for qPCR, and the data were analyzed using iCycler Thermal Cycler software (Applied Biosystems). Relative miRNA abundance was determined by normalizing to 5s rRNA using the 2Ct method.

### Protein analysis

Proteins were extracted from retinas and HREC as previously described [8]. The extracted proteins were quantified by using BioRad Protein DC Assay (Bio-Rad). Western blotting analysis was carried out as described [8] using specific antibodies for STAT3 phospho tyrosine 705 (pSTAT3; 1:2000; Cell Signaling, Danvers, MA) and TIMP3 (1:1000; Cell Signaling Technology, Beverly, MA), and corresponding secondary horseradish-conjugated antibodies (GE Healthcare, Pittsburg, PA). Phospho-STAT3 levels were normalized to total STAT3 (1:1000; Cell Signaling). Actin antibody was used as an internal control for TIMP3 expression (1:1000; Santa-Cruz Biotech). Chemiluminescence-based assay was used for protein detection (ThermoFisher, Rockford, IL).

### *In vitro* angiogenesis assay

Twenty-four-well plates were coated with Growth Factor Reduced Corning Matrigel Matrix (Corning Life Sciences, Tewksbury, MA) according the manufacture’s protocol. Twenty-four hours after transfection with *a.miR-21* or *s.amiR-21* cells were trypsinized, and 300 μl of the cell suspension containing 1.0 x 10^5^ cells was added to each well. The angiogenesis assay plate was incubated in hypoxic chamber (2% O_2_ at 37°C, 5% CO_2_) for 12 hours. In some experiments, cells were transfected with *miR-21* mimic or mimic *NC* and plated for angiogenesis assay for 12 hours at 37°C, 5% CO_2_ atmosphere. Captured digital images were analyzed for the extent of network formation by quantification of the number of interconnecting branching points.

### Statistical analysis

All data are presented as mean ± standard deviation (SD). The data were analyzed by Student t test or Mann-Whitney rank sum test using a computer-based software package (GraphPad Prism 6.0). P values less than 0.05 were considered significant.

## Abbreviations

RNV: retinal neovascularization
STAT3: signal transducer and activator of transcription 3
miR-21: microRNA-21
TIMP3: tissue inhibitor of matrix metalloproteinases 3
HREC: human retinal endothelial cells
OIR: oxygen-induced retinopathy

## References

[1] Rajappa M, Saxena P, Kaur J (2010). Ocular angiogenesis: mechanisms and recent advances in therapy. Adv Clin Chem.

[2] Chen J, Smith LE (2007). Retinopathy of prematurity. Angiogenesis.

[3] Sapieha P, Hamel D, Shao Z, Rivera JC, Zaniolo K, Joyal JS, Chemtob S (2010). Proliferative retinopathies: angiogenesis that blinds. Int J Biochem Cell Biol.

[4] Pahor D (1998). Visual field loss after argon laser panretinal photocoagulation in diabetic retinopathy: full- versus mild-scatter coagulation. Int Ophthalmol.

[5] Ali TK, El-Remessy AB (2009). Diabetic retinopathy: current management and experimental therapeutic targets. Pharmacotherapy.

[6] Hartnett ME (2015). Pathophysiology and mechanisms of severe retinopathy of prematurity. Ophthalmology.

[7] Penn JS, Madan A, Caldwell RB, Bartoli M, Caldwell RW, Hartnett ME (2008). Vascular endothelial growth factor in eye disease. Prog Retin Eye Res.

[8] Lamoke F, Labazi M, Montemari A, Parisi G, Varano M, Bartoli M (2011). Trans-Chalcone prevents VEGF expression and retinal neovascularization in the ischemic retina. Exp Eye Res.

[9] Caldwell RB, Bartoli M, Behzadian MA, El-Remessy AE, Al-Shabrawey M, Platt DH, Caldwell RW (2003). Vascular endothelial growth factor and diabetic retinopathy: pathophysiological mechanisms and treatment perspectives. Diabetes Metab Res Rev.

[10] Kowluru RA, Zhong Q, Santos JM (2012). Matrix metalloproteinases in diabetic retinopathy: potential role of MMP-9. Expert Opin Investig Drugs.

[11] Grant MB, Caballero S, Tarnuzzer RW, Bass KE, Ljubimov AV, Spoerri PE, Galardy RE (1998). Matrix metalloproteinase expression in human retinal microvascular cells. Diabetes.

[12] Di Y, Nie QZ, Chen XL (2016). Matrix metalloproteinase-9 and vascular endothelial growth factor expression change in experimental retinal neovascularization. Int J Ophthalmol.

[13] Huang H, Gandhi JK, Zhong X, Wei Y, Gong J, Duh EJ, Vinores SA (2011). TNFalpha is required for late BRB breakdown in diabetic retinopathy, and its inhibition prevents leukostasis and protects vessels and neurons from apoptosis. Invest Ophthalmol Vis Sci.

[14] Adamis AP (2002). Is diabetic retinopathy an inflammatory disease?. Br J Ophthalmol.

[15] Kern TS (2007). Contributions of inflammatory processes to the development of the early stages of diabetic retinopathy. Exp Diabetes Res.

[16] Li J, Wang JJ, Yu Q, Wang M, Zhang SX (2009). Endoplasmic reticulum stress is implicated in retinal inflammation and diabetic retinopathy. FEBS Lett.

[17] Mechoulam H, Pierce EA (2005). Expression and activation of STAT3 in ischemia-induced retinopathy. Invest Ophthalmol Vis Sci.

[18] Al-Shabrawey M, Bartoli M, El-Remessy AB, Ma G, Matragoon S, Lemtalsi T, Caldwell RW, Caldwell RB (2008). Role of NADPH oxidase and Stat3 in statin-mediated protection against diabetic retinopathy. Invest Ophthalmol Vis Sci.

[19] Bartoli M, Gu X, Tsai NT, Venema RC, Brooks SE, Marrero MB, Caldwell RB (2000). Vascular endothelial growth factor activates STAT proteins in aortic endothelial cells. J Biol Chem.

[20] Bartoli M, Platt D, Lemtalsi T, Gu X, Brooks SE, Marrero MB, Caldwell RB (2003). VEGF differentially activates STAT3 in microvascular endothelial cells. FASEB J.

[21] Bartoli M, Al-Shabrawey M, Labazi M, Behzadian MA, Istanboli M, El-Remessy AB, Caldwell RW, Marcus DM, Caldwell RB (2009). HMG-CoA reductase inhibitors (statin) prevents retinal neovascularization in a model of oxygen-induced retinopathy. Invest Ophthalmol Vis Sci.

[22] Mui AL (1999). The role of STATs in proliferation, differentiation, and apoptosis. Cell Mol Life Sci.

[23] Guinea-Viniegra J, Jimenez M, Schonthaler HB, Navarro R, Delgado Y, Concha-Garzon MJ, Tschachler E, Obad S, Dauden E, Wagner EF (2014). Targeting miR-21 to treat psoriasis. Sci Transl Med.

[24] Iliopoulos D, Jaeger SA, Hirsch HA, Bulyk ML, Struhl K (2010). STAT3 activation of miR-21 and miR-181b-1 via PTEN and CYLD are part of the epigenetic switch linking inflammation to cancer. Mol Cell.

[25] Escobar T, Yu CR, Muljo SA, Egwuagu CE (2013). STAT3 activates miR-155 in Th17 cells and acts in concert to promote experimental autoimmune uveitis. Invest Ophthalmol Vis Sci.

[26] Ambros V (2004). The functions of animal microRNAs. Nature.

[27] Esteller M (2011). Non-coding RNAs in human disease. Nat Rev Genet.

[28] Sundermeier TR, Palczewski K (2012). The physiological impact of microRNA gene regulation in the retina. Cell Mol Life Sci.

[29] Shen J, Yang X, Xie B, Chen Y, Swaim M, Hackett SF, Campochiaro PA (2008). MicroRNAs regulate ocular neovascularization. Mol Ther.

[30] Agrawal S, Chaqour B (2014). MicroRNA signature and function in retinal neovascularization. World J Biol Chem.

[31] Andreeva K, Soliman MM, Cooper NG (2015). Regulatory networks in retinal ischemia-reperfusion injury. BMC Genet.

[32] Zhou Q, Gallagher R, Ufret-Vincenty R, Li X, Olson EN, Wang S (2011). Regulation of angiogenesis and choroidal neovascularization by members of microRNA-23∼27∼24 clusters. Proc Natl Acad Sci U S A.

[33] Zhuang Z, Xiao Q, Hu H, Tian SY, Lu ZJ, Zhang TZ, Bai YL (2015). Down-regulation of microRNA-155 attenuates retinal neovascularization via the PI3K/Akt pathway. Mol Vis.

[34] Kovacs B, Lumayag S, Cowan C, Xu S (2011). MicroRNAs in early diabetic retinopathy in streptozotocin-induced diabetic rats. Invest Ophthalmol Vis Sci.

[35] Loffler D, Brocke-Heidrich K, Pfeifer G, Stocsits C, Hackermuller J, Kretzschmar AK, Burger R, Gramatzki M, Blumert C, Bauer K, Cvijic H, Ullmann AK, Stadler PF (2007). Interleukin-6 dependent survival of multiple myeloma cells involves the Stat3-mediated induction of microRNA-21 through a highly conserved enhancer. Blood.

[36] Zhou X, Ren Y, Liu A, Han L, Zhang K, Li S, Li P, Li P, Kang C, Wang X, Zhang L (2014). STAT3 inhibitor WP1066 attenuates miRNA-21 to suppress human oral squamous cell carcinoma growth *in vitro* and *in vivo*. Oncol Rep.

[37] Krichevsky AM, Gabriely G (2009). miR-21: a small multi-faceted RNA. J Cell Mol Med.

[38] Guduric-Fuchs J, O'Connor A, Cullen A, Harwood L, Medina RJ, O'Neill CL, Stitt AW, Curtis TM, Simpson DA (2012). Deep sequencing reveals predominant expression of miR-21 amongst the small non-coding RNAs in retinal microvascular endothelial cells. J Cell Biochem.

[39] Ragusa M, Caltabiano R, Russo A, Puzzo L, Avitabile T, Longo A, Toro MD, Di Pietro C, Purrello M, Reibaldi M (2013). MicroRNAs in vitreus humor from patients with ocular diseases. Mol Vis.

[40] Chen Q, Qiu F, Zhou K, Matlock HG, Takahashi Y, Rajala RV, Yang Y, Moran E, Ma JX (2017). Pathogenic role of microRNA-21 in diabetic retinopathy through downregulation of PPARalpha. Diabetes.

[41] Qing S, Yuan S, Yun C, Hui H, Mao P, Wen F, Ding Y, Liu Q (2014). Serum miRNA biomarkers serve as a fingerprint for proliferative diabetic retinopathy. Cell Physiol Biochem.

[42] Hu J, Ni S, Cao Y, Zhang T, Wu T, Yin X, Lang Y, Lu H (2016). The angiogenic effect of microRNA-21 targeting TIMP3 through the regulation of MMP2 and MMP9. PLoS One.

[43] Zhao Y, Xu Y, Luo F, Xu W, Wang B, Pang Y, Zhou J, Wang X, Liu Q (2013). Angiogenesis, mediated by miR-21, is involved arsenite-induced carcinogenesis. Toxicol Lett.

[44] Hewing NJ, Weskamp G, Vermaat J, Farage E, Glomski K, Swendeman S, Chan RV, Chiang MF, Khokha R, Anand-Apte B, Blobel CP (2013). Intravitreal injection of TIMP3 or the EGFR inhibitor erlotinib offers protection from oxygen-induced retinopathy in mice. Invest Ophthalmol Vis Sci.

[45] Qi JH, Ebrahem Q, Moore N, Murphy G, Claesson-Welsh L, Bond M, Baker A, Anand-Apte B (2003). A novel function for tissue inhibitor of metalloproteinases-3 (TIMP3): inhibition of angiogenesis by blockage of VEGF binding to VEGF receptor-2. Nat Med.

[46] Auricchio A, Behling KC, Maguire AM, O'Connor EM, Bennett J, Wilson JM, Tolentino MJ (2002). Inhibition of retinal neovascularization by intraocular viral-mediated delivery of anti-angiogenic agents. Mol Ther.

[47] Sabatel C, Malvaux L, Bovy N, Deroanne C, Lambert V, Gonzalez ML, Colige A, Rakic JM, Noel A, Martial JA, Struman I (2011). MicroRNA-21 exhibits antiangiogenic function by targeting RhoB expression in endothelial cells. PLoS One.

[48] Song B, Wang C, Liu J, Wang X, Lv L, Wei L, Xie L, Zheng Y, Song X (2010). MicroRNA-21 regulates breast cancer invasion partly by targeting tissue inhibitor of metalloproteinase 3 expression. J Exp Clin Cancer Res.

[49] Fujita S, Ito T, Mizutani T, Minoguchi S, Yamamichi N, Sakurai K, Iba H (2008). miR-21 gene expression triggered by AP-1 is sustained through a double-negative feedback mechanism. J Mol Biol.

[50] Caporali A, Emanueli C (2011). MicroRNA regulation in angiogenesis. Vascul Pharmacol.

[51] Handsley MM, Edwards DR (2005). Metalloproteinases and their inhibitors in tumor angiogenesis. Int J Cancer.

[52] Rundhaug JE (2005). Matrix metalloproteinases and angiogenesis. J Cell Mol Med.

[53] Lambert V, Wielockx B, Munaut C, Galopin C, Jost M, Itoh T, Werb Z, Baker A, Libert C, Krell HW, Foidart JM, Noel A, Rakic JM (2003). MMP-2 and MMP-9 synergize in promoting choroidal neovascularization. FASEB J.

[54] Stohr H, Anand-Apte B (2012). A review and update on the molecular basis of pathogenesis of Sorsby fundus dystrophy. Adv Exp Med Biol.

[55] Amour A, Slocombe PM, Webster A, Butler M, Knight CG, Smith BJ, Stephens PE, Shelley C, Hutton M, Knauper V, Docherty AJ, Murphy G (1998). TNF-alpha converting enzyme (TACE) is inhibited by TIMP-3. FEBS Lett.

[56] Janssen A, Hoellenriegel J, Fogarasi M, Schrewe H, Seeliger M, Tamm E, Ohlmann A, May CA, Weber BH, Stohr H (2008). Abnormal vessel formation in the choroid of mice lacking tissue inhibitor of metalloprotease-3. Invest Ophthalmol Vis Sci.

[57] Ebrahem Q, Qi JH, Sugimoto M, Ali M, Sears JE, Cutler A, Khokha R, Vasanji A, Anand-Apte B (2011). Increased neovascularization in mice lacking tissue inhibitor of metalloproteinases-3. Invest Ophthalmol Vis Sci.

[58] Gabriely G, Wurdinger T, Kesari S, Esau CC, Burchard J, Linsley PS, Krichevsky AM (2008). MicroRNA 21 promotes glioma invasion by targeting matrix metalloproteinase regulators. Mol Cell Biol.

[59] Abbott BP, Abbott R, Abbott TD, Abernathy MR, Acernese F, Ackley K, Adams C, Adams T, Addesso P, Adhikari RX, Adya VB, Affeldt C, Agathos M (2016). GW150914: the advanced LIGO detectors in the era of first discoveries. Phys Rev Lett.

[60] Liu LZ, Li C, Chen Q, Jing Y, Carpenter R, Jiang Y, Kung HF, Lai L, Jiang BH (2011). MiR-21 induced angiogenesis through AKT and ERK activation and HIF-1alpha expression. PLoS One.

[61] Ji R, Cheng Y, Yue J, Yang J, Liu X, Chen H, Dean DB, Zhang C (2007). MicroRNA expression signature and antisense-mediated depletion reveal an essential role of MicroRNA in vascular neointimal lesion formation. Circ Res.

[62] Jiang FS, Tian SS, Lu JJ, Ding XH, Qian CD, Ding B, Ding ZS, Jin B (2015). Cardamonin regulates miR-21 expression and suppresses angiogenesis induced by vascular endothelial growth factor. Biomed Res Int.

[63] Smith LE, Wesolowski E, McLellan A, Kostyk SK, D'Amato R, Sullivan R, D'Amore PA (1994). Oxygen-induced retinopathy in the mouse. Invest Ophthalmol Vis Sci.

[64] Connor KM, Krah NM, Dennison RJ, Aderman CM, Chen J, Guerin KI, Sapieha P, Stahl A, Willett KL, Smith LE (2009). Quantification of oxygen-induced retinopathy in the mouse: a model of vessel loss, vessel regrowth and pathological angiogenesis. Nat Protoc.

